# Chemical analysis of mineral trioxide agregate mixed with hyaluronic acids as an accelerant

**DOI:** 10.1590/0103-6440202305549

**Published:** 2023-12-22

**Authors:** Muthanna S. Ahmed, Nadia H. Hasan, Mohammed. G. Saeed

**Affiliations:** 1 Specialized dental center, Ninawa health directorate, Iraq.; 2 Department of Conservative Dentistry, College of Dentistry, University of Mosul, Mosul, Iraq.; 3 College of Veterinary, University of Mosul, Mosul, Iraq.

**Keywords:** MTA, hyaluronic acid, setting time, chemical properties, pulp

## Abstract

Mineral trioxide aggregate (MTA) has many clinical applications in dentistry; the main drawback is the long setting. The main objective is to investigate and compare the chemical effect of using two commercially available hyaluronic acid hydrogels (HA) instead of distilled water for mixing MTA as an accelerant of setting time.

**Materials and method:**

Test materials were divided into three groups; Group 1: (control) mixing MTA with distilled water supplied by the manufacturer; Group 2: mixing MTA with a hybrid cooperative complex of high and low molecular weight HA (Profhilo®); Group 3: mixing MTA with High molecular weight / non-cross-linked HA (Jalupro®). Mixing time, and setting time (initial and final) were determined, Fourier-transform infrared spectroscopy, Energy-dispersive X-ray spectroscopy, Field emission Scanning Electron Microscopy, and X-ray diffraction were performed.

**Results:**

mixing time, initial, and final setting time for (MTA + HA) groups were significantly different and lower in comparison to the control group (p < 0.05). This study revealed higher expression of calcium silicate hydrate and calcium hydroxide expression with higher Ca release in the MTA + HA group than the control group.

**Conclusion:**

commercially available HA demonstrated better chemical properties when used as a mixing medium for MTA. The Mixing and setting time for MTA + HA group were significantly shorter than those of the control group were. Thus, commercially available HA can be used as a mixing medium for MTA.

## Resumo

O agregado de trióxido mineral (MTA) tem muitas aplicações clínicas em odontologia, mas a principal desvantagem é a longa presa. O objetivo principal é investigar e comparar o efeito químico do uso de dois hidrogéis de ácido hialurônico (HA) disponíveis comercialmente em vez de água destilada para misturar o MTA como um acelerador do tempo de presa.

**Materiais e método:**

Os materiais de teste foram divididos em três grupos: Grupo 1: (controle) misturando o MTA com água destilada fornecida pelo fabricante; Grupo 2: misturando o MTA com um complexo cooperativo híbrido de HA de alto e baixo peso molecular (Profhilo®); Grupo 3: misturando o MTA com HA de alto peso molecular/não reticulado (Jalupro®). Foram determinados o tempo de mistura e o tempo de presa (inicial e final), a espectroscopia de infravermelho com transformada de Fourier, a espectroscopia de raios X com dispersão de energia, a microscopia eletrônica de varredura com emissão de campo e a difração de raios X.

**Resultados:**

o tempo de mistura, o tempo de presa inicial e final dos grupos (MTA + HA) foram significativamente diferentes e menores em comparação com o grupo de controle (*p* < 0,05). Esse estudo revelou maior expressão de silicato de cálcio hidratado e expressão de hidróxido de cálcio com maior liberação de Ca no grupo MTA + HA do que no grupo de controle.

**Conclusão:**

a HA disponível comercialmente demonstrou melhores propriedades químicas quando usada como meio de mistura para o MTA. O tempo de mistura e de presa do grupo MTA + HA foi significativamente menor do que o do grupo de controle. Portanto, a HA disponível comercialmente pode ser usada como meio de mistura para o MTA.

## Introduction

Direct pulp capping is a dental management that uses a dressing, medicament, or dental material to seal the exposed pulp when placed directly to protect and preserve the dental pulp vitality. Capping material stimulate pulp cells to generate reparative tertiary dentin ^1^. For many decades, calcium hydroxide considered as the material of choice among the different available pulp-capping agents ^2^. However, some of the limitations of using this technique include degradation on tooth flexure, dissolution by tissue fluids, and development of tunnel defects beneath dentinal bridges as a result of dissolution of the dressing that leaves voids beneath the restoration. The presence of such imperfection within the dentin in the form of tunnel defects will lead to microleakage, will cause bacterial re-infection, persisting pulp tissue inflammation and necrosis ^3^,^4^. Mineral trioxide aggregate (MTA) considered as the alternative gold standard to calcium hydroxide Ca(OH)_2_. It is available as a more effective direct pulp capping material than Ca(OH)_2_ ^5^,^6^,^7^. The composition of MTA is tricalcium silicate, tricalcium oxide, tricalcium aluminate, and silicate oxide ^8^. The effect of MTA is equivalent to that of Ca(OH)_2_ on the pulp tissue. In setting reaction of MTA, the primary product is Ca(OH)_2_ that will dissociate later on to give calcium and OH. The calcium ions act to enhance proliferation and attachment of progenitor cells from the pulp tissue. It also helps in the hard tissues formation due to dentinogenesis. In comparison to other capping materials, using MTA will lead to less tissue inflammation ^9^,^10^.

However, MTA has several drawbacks like high pH during setting that induce necrosis of the contacting tissue ^11^,^12^,^13^,^14^, sandy consistency that leads to difficulties in handling and placement, as well as its slow setting time which often requires another treatment session for the placement of final restoration ^15^.

 MTA produces complete dentin bridge formation after 2-3 months of its application as pulp capping material ^16^,^17^,^18^; however, several in vitro investigations used additives to the powder or the liquid in order to enhance the quality of the dentin bridge formation and inflammatory process after pulp capping using MTA and enhancing its physical properties, thus improving its clinical outcomes ^16^,^19^,^20^ such additives include Calcium Lactate Gluconate ^21^, propylene glycol ^22^, nano-titanium oxide (TiO_2)_, nano-zirconia, nano-silicon dioxide (SiO_2_), nano-aluminum oxide (Al_2_O_3_) ^23^, zinc oxide powder particles ^24^, Eggshell powder ^25^,tannic acid liquid ^26^,and chitosan ^27^.

Hyaluronic acid (HA) is an essential, naturally occurring compound that considers as a main component of the extracellular matrix (ECM) of the tissues in all adult animal. HA composed of glycosaminoglycan disaccharide that is consists of an alternately repeating units of D-glucuronic acid and N-Acetyl-D-glucosamine ^28^. Naturally, HA can be found in the skin, vitreous body, synovial fluid, joints, tendons, pleura, umbilical cord, and the pericardium. Fifteen grams of hyaluronic acid can be obtained from the body of a human weighing 70 kg ^29^. The interaction of HA with other macromolecules plays a major role in tissue morphogenesis, cell differentiation, migration, and adhesion ^30^. Which is used largely in many clinical applications and regenerative medicine because of its important biological properties such as biocompatibility, biodegradability, and non-immunogenicity ^31^.

The origin of commercial HA is from animal sources. In order to produce HA for medical applications, biotechnology and microbial fermentation techniques are used. Microorganism-derived HA is biocompatible with the mammalian body. In vivo, HA degrade rapidly by oxidative damage effect and the action of hyaluronidase enzymes ^32^. Chrepa et al. ^33^ showed the ability of commercial HA to stimulate stem cells of the apical papilla (SCAP) mineralization and odontogenic differentiation. It expressed significantly higher activity of alkaline phosphatase (ALP) with increased and upregulation of all genes related to odontoblastic differentiation like dentin sialophosphoprotein (DSPP), matrix extracellular phosphoglycoprotein (MEPE), and dentin matrix acidic phosphoprotein-1 (DMP-1). AlHowaish et al. ^34^ reported that commercially available HA has the ability to enhance vascularization within the pulp spaces and vascularized soft connective tissue formation with the presence of collagen fibers and fibroblasts within the spaces of the pulp.

The null hypothesis of this study states that hyaluronic acid can be used as a mixing media with MTA to enhance its chemical properties and reduce setting time. Therefore, we aimed to evaluate the chemical effects of HA, which when added to MTA, can reduce the setting time and enhance its chemical properties.

## Materials and method

The materials that were used in this study are reported in Box 1. MTA Repair HP was used in this study. Which has the same formula of conventional MTA but the radio pacifier used is calcium tungstate with the addition of a plasticizer agent to the mixing liquid ^35^. It has many clinical applications as a direct pulp capping material, root-end filling for retrograde restorations, pulpotomy, apexification, apexogenesis, and for root canal perforations repair. The manufacturer of MTA repair HP claim that the chemical properties of the original MTA is maintained in this formula, with improved physical property in relation to handling and manipulation.

The first HA used in this study was Profhilo^®^ (IBSANordic ApS, Denmark). It is a novel HA formula of stable hybrid cooperative complexes production technology (HyCoCos), representing an innovative thermal production process. Each Package contains one 2mL prefilled syringe containing 32 mg of high molecular weight HA (HMW HA) and 32mg of low molecular weight HA (LMW HA) dissolved in 2 mL of sodium chloride buffered physiological solution. The process of HA production starts with a simple mixture of 32mg of HMW HA weights (1100 -1400kDa) and 32mg of LMW HA weights (80 - 100kDa). The hybrid crosslinking technology stabilizes the mixture using innovated thermal process. No chemicals or crosslinking agents are used, as the crosslinking is performed thermally in two steps: a high-temperature step, and a low-temperature step.

The second HA used in this study was Jalupro^®^ HMW (PROFFESSIONAL DERMA SA, ITALY), an HMW, non-cross-linked HA. The Jalupro^®^ HMW comprises disposable syringe of a sterile sodium hyaluronate gel (20 mg/ml gel of 1200-1400 kDa).

The following characteristics were evaluated in included study: mixing time (*n*=5), initial setting time (*n*=5), final setting time (*n*=5), Fourier-transform infrared spectroscopy (FTIR) (*n*=1), X-ray diffraction (XRD) (*n*=1), Field emission Scanning Electron Microscopy (FESEM) (*n*=1), and Energy - dispersive X-ray spectroscopy (EDAX) (*n*=1).

**Box:**
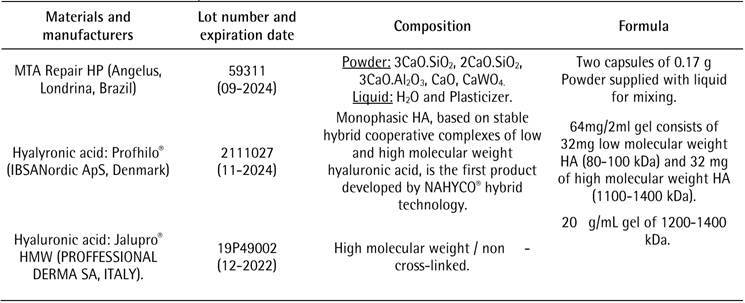
1. materials used in the study.

### Samples preparation

To evaluate the changes in the chemical properties of MTA after mixing it with HA, the Ex-vivo study included the following groups:

Group 1 (control): MTA + distilled water.

Group 2 (HA1): MTA + Profhilo^®^ HA (LMW HA + HMW HA).

Group 3 (HA2): MTA + Jalupro^®^ HA (HMW non cross-linked).

To prepare the hydrated cement samples, the MTA powder was mixed with the supplied distilled water for control group, and with hyaluronic acid (profhilo^®^, Jalupro^®^) for group 2 and 3 respectively. The powder and the liquid were dispended on a glass slab and mixed with a metal spatula for manual mixing. A paste with a homogeneous consistency was obtained. We followed the manufacturer instructions for the powder/liquid ratio and mixing protocol.

After preparing study groups, the following chemical tests were conducted:

### Mixing time

The mixing time was evaluated according to the specifications of the American National Standards Institute/American Dental Association ANSI/ADA Standard No. 96 (2020) by using three samples for each group (*n*=3). The cement was prepared in accordance with the manufacturer’s instructions by mixing the by mixing 0.17 g of powder to 2 drops of liquid to ensure that the preparation of each specimen is completed from one mix. The mixing liquids were (the supplied distilled water supplied by the manufacturer, HA1, HA2). A metal spatula used for mixing on a glass slab to form a paste of putty-like consistency. The test was performed under controlled environments (37±1C and 95±5% humidity). A fresh mix has been prepared for each specimen.

### Setting time (initial and final settings)

The initial and final setting times were evaluated according to the criteria and conditions specified by both the (ANSI/ADA) specification No. 96-2020 and the American Society for Testing and Materials (ASTM: C266 - 21) methods (which represents the use of Gillmore needles to determine the setting time of hydraulic-cement paste). Three samples (*n*=3) were used for each group.

For the initial setting time measurement, one of Gillmore needles, which is of 2.12 mm in diameter, and 113.4 g weight is used. While the second needle which is of 1.06 mm in diameter, and 453.6 g weight is used for final setting time measurement.

The test was performed under controlled temperature and relative humidity (37±1C and 95±5% humidity), After mixing the cement with the proposed mixing liquid, 5 cylinder-shaped stainless steel mold (*h*= 5 mm and *d*= 10 mm) were filled with the material while it is in a plastic condition. To perform the test, the tip of the penetrometer was lowered vertically to touch the surface of the samples and left it in place for 5 seconds. The needle of the penetrometer was lowered until it stopped making circular indentations in the cement sample when examined using 2.5X magnification loops. The setting time was described as the time elapsed between the end of the mixing process and the time when the penetrometer needle stopped making circular indentation on the surface of the prepared cement samples ^36^.

### FTIR

Changes in the functional groups of the proposed mixing media (HA) and the MTA powder before and after mixing were evaluated using FTIR analysis. Infrared spectra records between 400 and 4000 cm^-1 (^ ^37^.

FTIR analysis was performed using ALPHA FTIR (Bruker, Germany). As prior preparation of the samples was not required; thus, the samples were placed directly on the diamond crystal for examination 48 hours after the material had set completely.

### XRD

XRD analysis was performed using a PAN alytical X’Pert PRO diffractometer (Almelo, Netherlands), with Cu-Kα radiation (0.154187 nm). The diffractometer was operated at 45 kV and 40 mA using a step size of 0.02 and a 500 s exposure time. Phase identification was accomplished using search-match software using XPERT high score software.

### FESEM / EDAX

The morphologies and microstructure with surface analysis of the mixed cement were examined using FESEM (TECSCAN FEG SEM MIRA 3LMU, Czech Republic). FESEM incorporated a cold cathode field emission gun that operates with a 0.5-30 kV voltage range.

EDAX analysis done for the same samples as the x-ray cone is coupled with the SEM device.

### Statistical Analysis

Statistical analysis of the collected data was performed using SPSS (version 22.0; IBM-SPSS Inc., Chicago, IL, USA) package program with analysis of variance (ANOVA) and Tukey post hoc test. A *p* value of less than 0.05 considered as statistically significant.

## Results

### Mixing time

**Figure 1:**
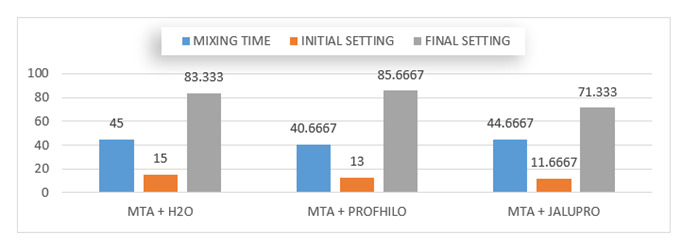
Column graph for Duncan^’^s multiple range test showed the mixing time; initial setting; and final setting time for the tested materials.

The mixing time for both HA groups were lower than that for the control group; HA1 (40.66 s), HA2 (44.66 s), and control group (45 s) as shown in Table 1 and Figure 1.

**Table 1:** One - way (ANOVA) showed the effect of different mixing medium on the mixing time of MTA (per second).

ANOVA	Sum of Squares	Mean Square	F	***P-* value**
Between Groups	34.889	17.444	31.400	0.001
Within Groups	3.333	0.556		
Total	38.222			

### Setting time

The initial setting time for both HA groups were lower than the control; control group (15 minute), HA1 group (13 minute), and HA2 group (11.66 minute) as shown in Figure (1).

The final setting time of the HA2 groups was lower than that of the control group and HA1 group; control group (83.33 minute), HA1 group (85.66 minute), and HA2 group (71.33 minute) as shown in Figure (1).

**Table 2:** One - way (ANOVA) showed the effect of different mixing medium on the initial setting time of MTA (per minute).

ANOVA	Sum of Squares	Mean Square	F	***P-* value**
Between Groups	16.889	8.444	19.000	0.003
Within Groups	2.667	0.444		
Total	19.556			

**Table 3:** One - way (ANOVA) showed the effect of different mixing medium on the final setting time of MTA (per minute).

ANOVA	Sum of Squares	Mean Square	F	***P-* value**
Between Groups	354.889	177.444	44.361	0.000
Within Groups	24.000	4.000		
Total	378.889			

### FTIR analysis

*FTIR analysis for the hydrating media*

**Figure 2:**
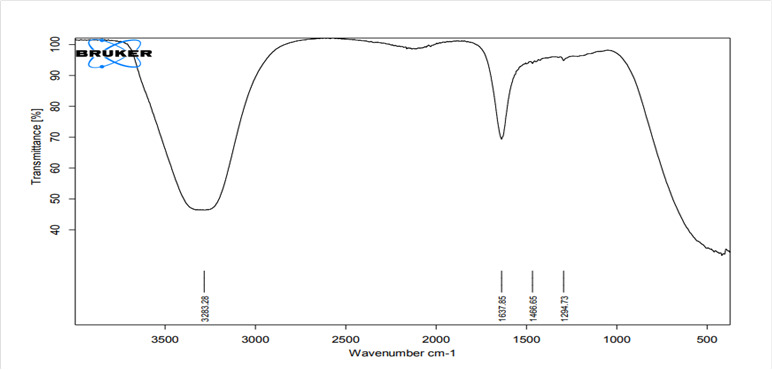
FTIR spectra of the mixing liquid supplied by the manufacturer.

FTIR spectra of liquids (distilled water, HA1, HA2) of all investigated hydration media shown in Figures 2, 4, and 6 revealed broad bands corresponding to the OH group spectra of water molecules found at ≈3300 cm^−1 (^ ^38^,^39^, and bands at 1637 cm^−1^ that can be assigned to the OH bending mode of absorbed water, overlapping the C = O group. The band at 1637 cm^−1^ can be assigned to water molecules which is associated to sulfate (gypsum) phase ^40^.

**Figure 3:**
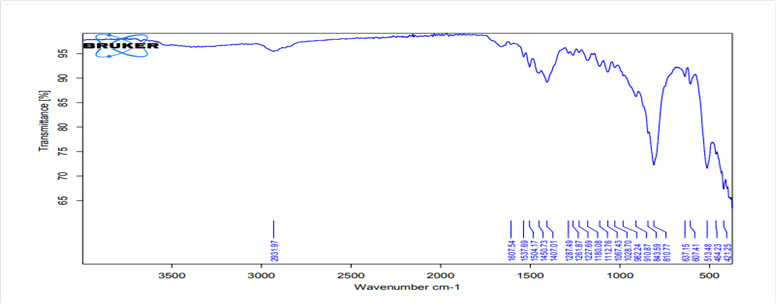
FTIR spectra of the MTA powder mixed with the supplied liquid.

FTIR analysis of the mixed samples in Figures 3, 5, and 7 showed Methyl spectra band (C-H) at 2980-2870 cm^−1^ in all groups. Carbonate bands (C-O) were observed at 1460-1420 and 1240-1200 cm^−1^. Unlike the liquid used for mixing the MTA powder, the absorption band corresponding to O-H stretching was not prominent for the control or HA3 groups, whereas it was at 3361.11 cm^-1^ for HA2 group. Aragonite (CO_3_^-2^) bands revealed antisymmetric stretching and located at 1450.7-1499.55 cm^-1^. The carbonation of the hydrated phases by the effect of atmospheric Co_2_ possibly caused these bands. They were slightly larger for samples prepared with HA2 solution because of the accelerated rate of early hydration reactions, the formation of more hydrated compounds, and the release of more free Ca(OH)_2_ in the reaction medium. In the set material, alite and belite phases showed a band at ≈ 513,437 cm^−1^ representing the anhydrate calcium silicate of both phases.

**Figure 4:**
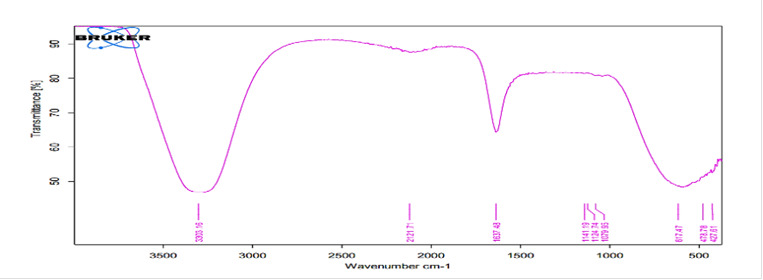
FTIR spectra of stable hybrid cooperative complexes HA.

**Figure 5.**
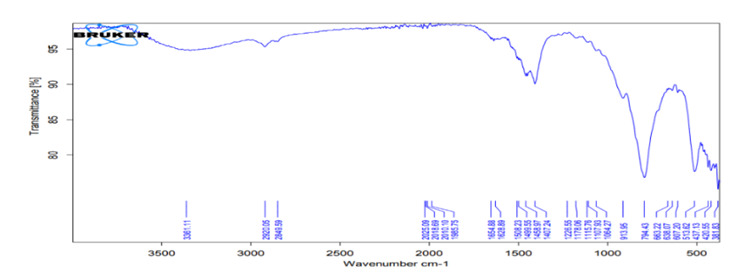
FTIR spectra of the MTA powder mixed with stable hybrid cooperative complexes HA.

**Figure 6:**
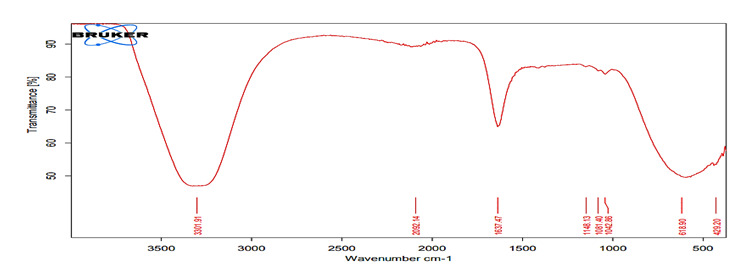
FTIR spectra of non cross-HA.

**Figure 7:**
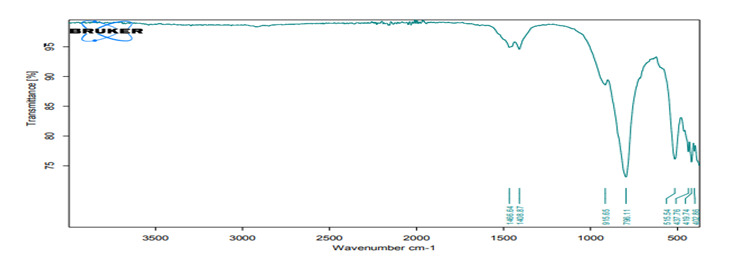
FTIR spectra of the MTA powder mixed with non cross-linked HA

**Figure 8:**
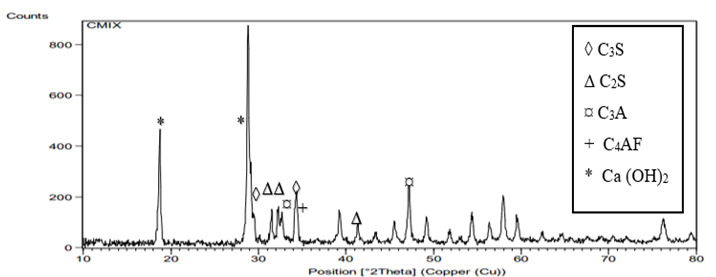
XRD analysis of MTA powder mixed with the liquid supplied by the manufacturer.

**Figure 9:**
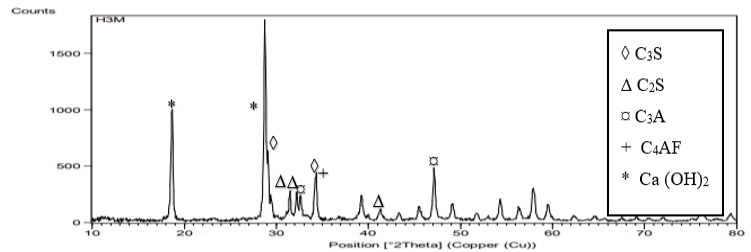
XRD analysis of MTA powder mixed with stable hybrid cooperative complexes HA.

**Figure 10:**
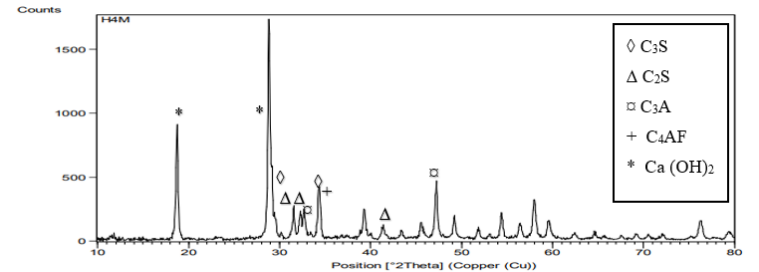
XRD analysis of MTA powder mixed with non-cross-linked HA.

Graphical representations of the XRD spectra are presented in Figures 8,9,10. All groups showed peaks at 29.3^ο^,34.4 = 2*θ* representing tricalcium silicate (C_3_S). Peaks representing dicalcium silicate (C_2_S) was observed at 2*θ* = 32.45^ο^,31.9, and at 41.66^ο^. Other peaks representing tricalcium aluminate (C_3_A) were recorded at 33.1^ο^ and 47.62 with peak of tetracalcium aluminoferrate (C_4_AF) observed at 34.26^ο^. All samples revealed Ca(OH)_2_ at peaks 28.69 and 18. The peaks of Ca(OH)_2_ for the HA groups were higher (nearly twice) than those of the control group.

### Morphologies and microstructural analysis with (FESEM)

**Figure 11:**
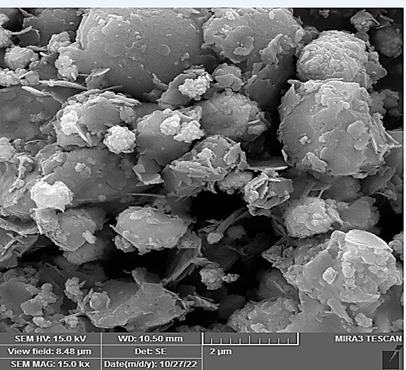
FESEM of MTA mixed with the supplied liquid by the manufacturer (X 15000).

**Figure 12:**
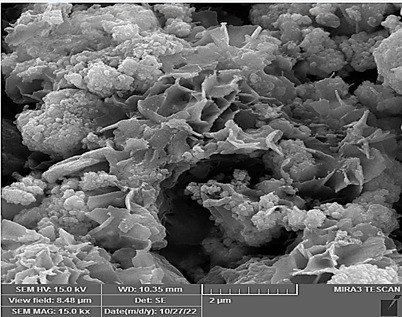
FESEM of MTA mixed with stable hybrid cooperative complexes HA (X 15000).

**Figure 13:**
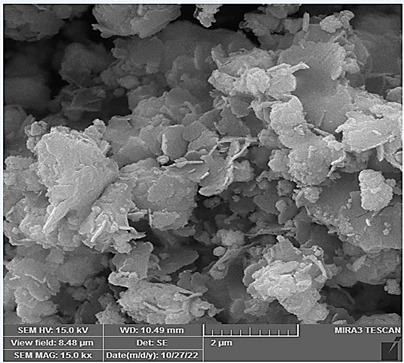
FESEM of MTA mixed with HA (X 15000).

**Figure 14:**
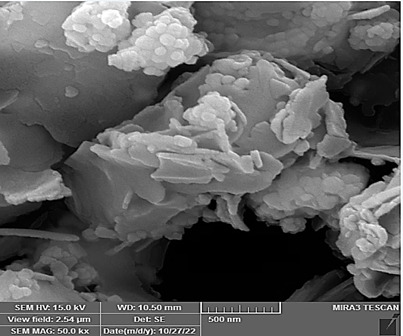
FESEM of MTA mixed with the supplied liquid by the manufacturer (X 50000).

**Figure 15:**
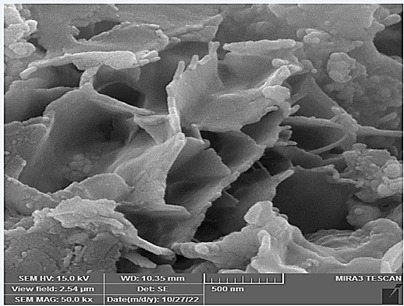
FESEM of MTA mixed with stable hybrid cooperative complexes HA (X 50000).

**Figure 16:**
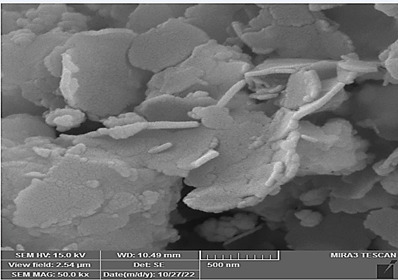
FESEM of MTA mixed with HA (X 50000).

At higher magnifications (Figures11,12,13,14,15,16), spiky-ball like clusters or clusters like structure lie under an amorphous layer with needle- like crystals and plates projecting out on its periphery with an irregular shape and severe agglomeration of small particles, which can be observed as small irregular particles interspersed. Calcium silicate hydrate (CSH) exhibit a typical honeycomb pattern.

### EDAX analysis

EDAX analysis show the elemental peaks for each material. The same elements are shown EDAX analysis showed elemental peaks for each material. The same elements were observed in all groups of tested materials (calcium, aluminum, oxygen, silica, and carbon).

The percentages of calcium production by weight and the atomic weight was higher in group 3 than in group 1 (control) and 2.

### EDAX analysis results of group 1 (MTA + distilled water)

**Figure 17:**
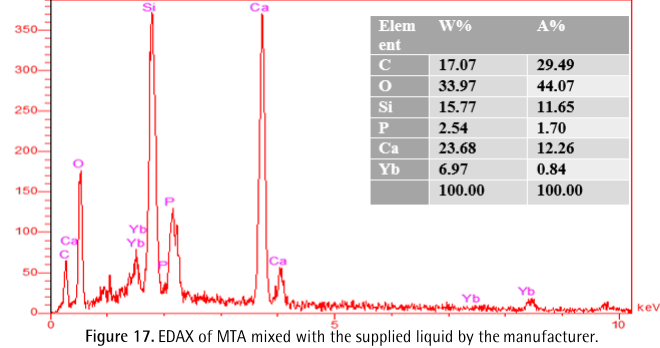
EDAX of MTA mixed with the supplied liquid by the manufacturer.

From the EDAX analysis of control group (Figure 17), C (17.07%W) (29.49%A), Si (15.77%X) (11.65%A), P (2.54%W) (1.70%A), and Ca (23.68%W) (12.26%A) were discovered.

### EDAX analysis results of group 2 (MTA + HA1)

**Figure 18:**
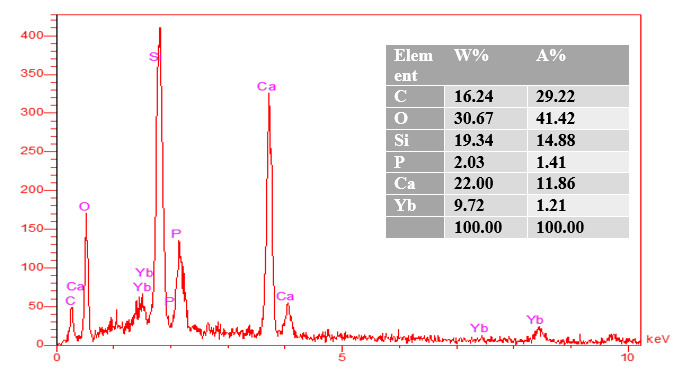
EDAX of MTA mixed with stable hybrid cooperative complexes HA.

From the EDAX analysis of HA1 group (Figure 18), C (16.24%W) (29.22%A), Si (19.34%X) (14.88%A), P(2.03%W) (1.41%A), and Ca (22.00%W) (11.86%A) were discovered.

### EDAX analysis results of group 3 (MTA + HA2)

**Figure 19:**
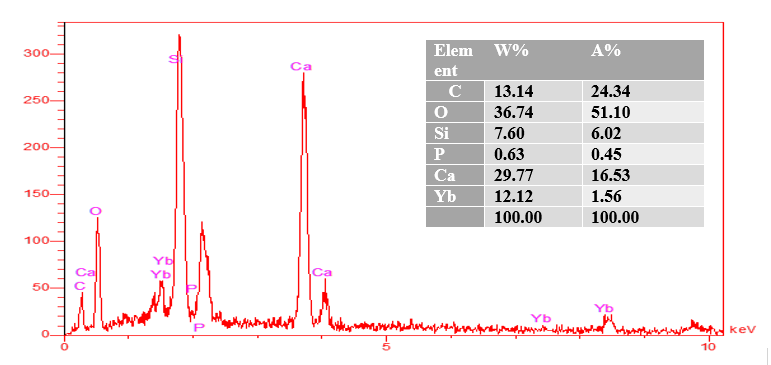
EDAX of MTA mixed with non cross-linked HA.

From the EDAX analysis of control group (Figure 19), C (13.14%W) (24.34%A), Si (7.60%X) (6.02%A), P(0.63%W) (0.45%A), and Ca (29.77%W) (16.53%A) were discovered.

## Discussion

To our knowledge, no study (to date) has evaluated the chemical properties of MTA mixed with commercially available HA as a mixing liquid. As MTA has long setting time, many additives has been evaluated to reduce its setting time and enhance its chemical properties. The chemical effects of hybrid cooperative complex HA and HMW HA that, when added to MTA was evaluated in this study and showed that they can reduce the setting time and improve the chemical properties of MTA.

The tissue repair mechanism in vital pulp therapy involves the replacement of the damaged and destroyed odontoblasts with newly redeveloped odontoblasts-like cell that originate from human dental pulp stem cells (HDPSCs) in non-injured sites of the pulp ^41^. However, applying the pulp capping procedure when dental caries are involved is challenging and must be limited to only a few cases following fine case election criteria ^42^,^43^,^44^. A limitation of this procedure is the severe inflammatory reactions induced by synthetic pulp capping materials. When inflammation develops, the integrity of the newly formed dentin-like bridge is disrupted, which increases microbial recontamination and results in secondary pulpal inflammation. In addition, the blood supply in the axial wall of the exposed pulp tissue is blocked, leading to necrosis of the pulp tissue. Complete retreatment is mandatory in case where dentin bridge formation may occur within the pulp but inflammation is irreversible ^45^.

HMW HA exhibits anti-inflammatory effect by controlling the inflammatory cells recruitment ^46^ ^,^ ^47^ and reduce the production of inflammatory cytokines by multiple cell types ^48^ ^,^ ^49^, In addition, HMW HA interacts with the cluster of differentiation 44 (CD44) receptors found on the surface of monocytes and granulocytes cells ^32^. The interaction between HMW HA and CD44 can affect different intracellular signaling pathways that control biological processes such as cell proliferation, migration, and adhesion to ECM components, angiogenesis ^50^,^51^,^52^,^53^, the intracellular reactive oxygen species (ROS) elimination ^54^, as well as reduction of the damage happening in deoxyribonucleic acid (DNA) ^55^,^56^. Accordingly, it can be used as a good candidate to replace the distilled water as a mixing medium for MTA with better inflammatory modulation properties.

HA seems to have stimulatory and enhancing effect on the vascularization of the affected tissues, probably by direct effect on endothelial cells ^57^. While indirectly, it has the ability to create conditions that favors tissue mineralization. It stimulates the migration of different types of cells into the injured area, followed by proliferation and differentiation of the migrated cells into odontoblast-like cells ^58^. BOGOVIĆ et al. ^59^ reported that cultures of pulp tissue treated with HA showed a larger number of odontoblasts and fibroblasts differentiation than cultures treated with Ca(OH)_2_, which indicates that HA has greater potential for reparative dentin formation. In cultures treated with HA, smaller proportion of necrotic and apoptotic cells were found. Viability analysis has also shown that HA is more biocompatible and less toxic to pulpal tissues than Ca(OH)_2_.

The results of the mixing time and setting time (initial, final) of both tests revealed a significant difference between the HA groups (group 2, group 3) and the control group, with the lowest initial and final setting time was for group 3 (non-cross-linked HA). The initial setting time for group 1 was (15.00 ± 1.00) minute, the initial setting time for group 2 was (13.00 ± 0.00) minute and for group 3 was (11.66± 0.55) minute. The final setting time for group 1 was (83.33 ± 2.08) minute, group 2 was (85.66 ± 1.15) minute, and for group 3 (71.33 ± 1.52) minute. These results indicate that hybrid cooperative complex HA and HMW HA can be used as an accelerant for MTA. The null hypothesis for the setting time was accepted based on the results obtained from this study.

The process of MTA setting reaction is complex. First, the C_3_S particles react with OH group of water. In this process, the margins of the powder of C_3_S is dissolved to form CSH ^60^. The main composition of CSH is calcium and silicon which were derived from MTA and OH ion which is derived from the liquid used to be mixed with the MTA powder. The other product of MTA setting reaction is Ca(OH)_2_, which is known to be formed as a byproduct of the process of MTA hydration reaction ^61^. The elevated pH of MTA after mixing can be explained as a result of Ca(OH)_2_ production. It was also hypnotized that Ca(OH)_2_ formation is the precursor of the ability of MTA to produce hard tissue. As the setting reaction further progresses, the formed Ca(OH)_2_ reacts with calcium sulfate to produce ettringite ^11^. The reaction of calcium hydroxide with phosphate ion produce amorphous calcium phosphate, which eventually give rise to hydroxyapatite ^62^. Later on, Ca(OH)_2_ dissociate to give calcium ions which stimulates the differentiation of progenitor cell in order to repair the damaged dental hard tissues ^63^ and hydroxyl ions (OH) which has an antimicrobial property ^64^.

FTIR chemical tests were performed 48 h after mixing of the MTA, whereas (XRD, FESEM, EDAX) were performed 28 days after mixing as hydration process of C_3_S is known to be slow. Therefore, complete hydration of the C_3_S requires 28 days, which makes the setting time of MTA long ^65^. After mixing the MTA with distilled water, the hydration of C_3_S phase starts immediately and its paste will solidify to a more hardened structure. About 90% of the anhydrous C_3_S phase will be hydrated at ambient temperature and curing age of 28 days, C_3_S phase is considered the main constituent in MTA material that is responsible for development of mechanical strength by production of calcium silicate hydrate (C-S-H) ^66^,^67^. At early hydration periods (3 and 7 days), faster hydration reaction rate of C_3_S phase was detected which liberate an excess amounts of Ca(OH)_2_. Causing an elevation in the pH of the hydration medium to more alkaline (pH˃8). At later ages of hydration (28 days), large amounts of C-S-H gel is formed as a hydrated compound of tri-calcium silicate material that will encapsulate the anhydrous C_3_S particles by filling the micro-pores of the hardened structure. Preventing more water molecules penetration that is needed for the hydration process progression ^68^,^60^. At later hydration ages (7 and 28 days), this covering layer will be distorted and the water molecules will come in contact with the anhydrous C3S grains ^68^.

The FTIR spectra of the mixed media (distilled water, hybrid cooperative complexes HA, non-cross-linked HA) were the same. The most important spectrum is that of the OH group involved in the hydration reaction (band at 3600-3000 cm^−1^) ^69^. The most likely reactions for HA are involve OH groups ^70^. For the set material, the OH band was prominent in the FTIR spectra of the completely set materials, indicating the formation of hydrated phases and their byproducts (Ca(OH)_2_, CSH) ^71^.

HA can be depolymerized using both alkaline and acidic hydrolysis. Dilute and semi- dilute solutions of HA are degraded with faster degradation rate at high pH ^72^. The pH of MTA increases after mixing; thus, the alkaline media enhance HA hydrolysis and release of OH.

For HA groups, especially for group 3 (non-cross-liked HA), the OH band is nearly disappeared which indicates maximum integration of the OH group into the hydration reaction and could produce higher hydration reaction byproducts (CSH, Ca(OH)_2_). This concept can be confirmed clearly from the higher band of Ca(OH)_2_ for HA group 3 by XRD analysis in which the resulted spectrum of Ca(OH)_2_ at the peak 28.69 and 18 for HA groups is as twice as that of the control group which indicates higher production rate of Ca(OH)_2_ when using HA as a hydrating media instead of distilled water.

The higher diffraction peak of CSH at 2*θ* = 29.3° for HA group content in the final hydrated product will give a faster and better hydration reaction in comparison to the control group, with a shorter setting time ^73^. The same results can be confirmed when examining the EDAX data that showed higher Ca release for non-cross-linked HA group (29.77%W,16.35%A) in comparison to the control group.

FESEM study of both HA groups reveals calcium phosphate/HA (CaP/HA) composite material suggested to be consist of CaP nanoparticles that shows a uniform distribution mode throughout the matrix of HA-MTA. Previously, it has been reported that HA can serve as a template or scaffold for CaP crystals growth that controls their morphology and size like in the process of native bone biomineralization. Chen et al. ^74^ showed that HA inhibits agglomeration of CaP crystals. Inhibition was initiated by first entrapping Ca into the matrix of HA due to the complexing interactions and then surrounding newly formed CaP crystals into HA loops. Based on the results of this study, the null hypothesis regarding the enhancement of the chemical properties of MTA is accepted.

However, this study has several limitations, starting from the selection of the HA type for the study as there are many types that are commercially available (hybrid cooperative complex HA, non-cross-linked HA, chemically cross-linked HA). Further *in-vivo* studies are needed to evaluate the effect of HA on inflammatory process that is a part of the pulp tissue healing process.

## Conclusion

HA can be used for mixing MTA with shorter setting time, higher Ca ion release, and higher CSH and Ca(OH)_2_ production. When used as a mixing media for MTA, commercially available HA revealed better chemical properties, with shorter setting time, and higher Ca ion release. It is readily available with no previous preparation steps. Thus, it suggests that commercially available HA can be used as a mixing medium for MTA.
